# Puerperal Eclampsia

**Published:** 1900-11-17

**Authors:** 


### PUERPERAL ECLAMPSIA.

Dr. Killebrew,[1] giving his views as to the treatment of puerperal eclampsia, emphasises the advantage of normal salt solution given as a prophylacitic in the form of high enemata of from one to three pints once or twice every day, and for treatment in the form of an intravenous injection. The saline given as a high enema is absorbed, he says, by the mucous membrane of the colon, enters the circulation, dilutes the blood and the toxins contained therein, and by stimulating the skin and kidneys to greater activity causes an increased elimination of the poison, whatever it be, which is the cause of the disease. If convulsions should have already occurred, he places the means to be employed in the following order:—(1) Control convulsions with chloroform; (2) bleed the patient and give intravenous injection of normal salt solution; (3) empty the uterus.

Most practitioners, he says, would empty the uterus before resorting to the saline; but emptying the uterus may take two hours, or even more, whereas the infusion of normal saline can be given in a few minutes. This is a matter of importance, for in some cases the toxæmia is so profound when the patient is first seen that she will be dead before the uterus can be emptied. It therefore seems better to give the infusion of salt solution first, and allow it to be doing its work while the uterus is being emptied. After these measures have been taken the colon should be thoroughly irrigated.

[1] New York Medical News, Nov. 3.

